# The Annual Circular of the Baltimore College of Dental Surgery

**Published:** 1869-04

**Authors:** 


*The Annual Circular of the Baltimore College of Dental Surgery.*—The *Thirtieth* Annual Circular of this College has just been issued, containing the Course of Study, Statutes, Terms, &c., with the names of the graduates and matriculants of the Session of 1868-69. The number of matriculants during the past session was *sixty-four*, representing fifteen States and Europe; fifty-five from the Southern States, seven from the Northern States and two from Europe.

The Faculty, among other additions to the extensive College collection, have added a costly *Manikin* of life size, made in Paris, which has been greatly admired by all who have visited the institution during the past session.

Several changes have occurred in the Faculty during the past year, and it is now composed of the following gentlemen: Thomas E. Bond, M.D. Prof. of

Pathology and Therapeutics; Philip H. Austen, M.D., D.D.S., Prof. of Dental Science and Mechanism; F. J. S. Gorgas, M.D., D.D.S., Prof. of Dental Surgery; Henry R. Noel, M.D., Prof. Comparative and General Pathology, Dietetics and Hygiene; E. Lloyd Howard, M.D., Prof. of Histology, Comparative, Descriptive and Pathological Anatomy; Russell Murdoch, M.D., Prof. of Chemistry; H. M. Grant, M.D., D.D.S., Demonstrator of Operative Dentistry, and T. S. Waters, D.D.S., Demonstrator of Mechanical Dentistry. Dean of the College, F. J. S. Gorgas, 259 N. Eutaw St.

The *Thirtieth Annual Session* will commence on the *fourteenth* of October, 1869, and continue until March 1870. The first two weeks of the Session are devoted to Infirmary Practice, Demonstrations and Preliminary Lectures. The Regular Lectures commence on the first of November.

